# Store-Independent Calcium Entry and Related Signaling Pathways in Breast Cancer

**DOI:** 10.3390/genes12070994

**Published:** 2021-06-29

**Authors:** Mohamed Chamlali, Lise Rodat-Despoix, Halima Ouadid-Ahidouch

**Affiliations:** Laboratory of Cellular and Molecular Physiology, UR-UPJV 4667, University of Picardie Jules Verne, 80000 Amiens, France; mohamed.chamlali@etud.u-picardie.fr (M.C.); lise.despoix@u-picardie.fr (L.R.-D.)

**Keywords:** calcium channels, basal calcium entry, Orai-K^+^ channel complex, transient receptor potential channels, voltage-gated calcium channels, breast cancer

## Abstract

Known as a key effector in breast cancer (BC) progression, calcium (Ca^2+^) is tightly regulated to maintain the desired concentration to fine-tune cell functions. Ca^2+^ channels are the main actors among Ca^2+^ transporters that control the intracellular Ca^2+^ concentration in cells. It is well known that the basal Ca^2+^ concentration is regulated by both store-dependent and independent Ca^2+^ channels in BC development and progression. However, most of the literature has reported the role of store-dependent Ca^2+^ entry, and only a few studies are focusing on store-independent Ca^2+^ entry (SICE). In this review, we aim to summarize all findings on SICE in the BC progression field.

## 1. Introduction

Cancers are a major public health problem due to their incidence and, more particularly, their mortality. Among all cancers, breast cancer (BC) is one of the most diagnosed in the world. Despite significant discoveries in treatment, some BC are currently incurable. Thereby, we still need to identify targets to treat BC. In recent years, a research field has developed around the role of ion channels and their implication in tumor progression [1]. The involvement of ion transporters in tumor development could thus classify cancers as onco-channelopathies [2]. Furthermore, several studies reported the involvement of calcium (Ca^2+^) channels in almost all hallmarks of cancer [3]. Research on BC has shown the involvement of a certain number of proteins related to its development and progression. It has also been found that Ca^2+^-regulating proteins are key effectors in BC. Indeed, Ca^2+^ is recognized as a universal intracellular second messenger involved in a plethora of physiological as well as physiopathological processes, such as cell proliferation, migration, invasion, apoptosis, and chemoresistance in a cancer situation [4,5]. Many studies have already clarified the role of Ca^2+^ signaling in cancer and especially BC, suggesting its importance in the altering processes that shape cancer cells [6]. However, calcium transport and its regulation remain to be completely understood.

Several pathways, with different modes of action, permit a Ca^2+^ influx. The most known is the store-operated Ca^2+^ entry (SOCE) pathway which is regulated by the activation of the Ca^2+^ release-activated channels encoded by Orai channels via stromal interaction molecule (STIM) proteins [7]. It is well known in the literature that these channels play critical roles in carcinogenesis, including BC [8]. However, recent studies have reported the role of store-independent Ca^2+^ entry (SICE) in regulating BC processes and several hallmarks of BC. Indeed, SICE is known to be induced either by arachidonic acid (AA) and/or the AA metabolite leukotriene C4 (LTC4) pathways and regulated by both Orai1 and Orai3 channels [9], or by the functional coupling between Orai1 and secretory pathway Ca^2+^-ATPase (SPCA2) [10]. In addition to these two major pathways, SICE can also be mediated by voltage-gated Ca^2+^ channels (VGCC), mechanosensitive Ca^2+^ channels, ligand-activated Ca^2+^ channels, as well as constitutively activated Ca^2+^ channels [11,12,13].

Several reviews have focused on the description and role of SOCE through the stromal interaction molecule (STIM) protein activation [8,14]. However, studies on SICE remain elusive. In this review, we aimed to summarize the different studies which have shown the role of SICE in the regulation of BC processes.

## 2. Orai Channels

Orai channels play distinct roles in different BC subtypes [15]. Two isoforms (Orai1 and Orai3) in particular have been found overexpressed in BC. Regarding Orai3, it was found overexpressed in 76.9% of 13 tested BC samples when compared to non-tumoral breast ones [16]. In addition, a positive correlation between Orai3 and the c-Myc proto-oncogene transcriptional expression in BC tissues has also been reported [17]. By analyzing Orai3 in clinical BC samples through the analysis of a public dataset, Hasna et al. proposed Orai3 as a predictive marker in the resistance to chemotherapeutic drugs [18]. Subsequently, a study conducted by Azimi et al. reported a sensitivity of Orai3 to hypoxia [19]. They observed an increase in Orai3 expression in response to hypoxia in both basal and luminal types of BC cells, and identified hypoxia and hypoxia-inducible factor 1 α (HIF1α) as critical regulators of Orai3 expression in these types of cell lines [19]. Finally, Orai3 transcriptional expression is regulated via the expression of micro-ribonucleic acid (miRNA). Indeed, it has been shown that miR34A and miR18A/B inhibit and activate Orai3 expression, respectively [20]. On the other hand, Orai1 was found expressed in the mammary gland and its expression increased during lactation assuming the trans-epithelial Ca^2+^ transport [21]. Orai1 was also found up-regulated in BC cell lines and is particularly highly expressed in basal subtype cells where it regulates migration [19,21,22].

Interestingly, an association between Orai channels and transient receptor potential canonical 6 (TRPC6) channels has been reported. Thus, both Orai1 and Orai3 plasma membrane localization is allowed by TRPC6 in BC. Indeed, TRPC6 calcium activity permits these Orai isoforms to be translocated to the plasma membrane and thus participates in a novel way to modulate the Ca^2+^ influx in BC [23].

Several studies have shown an activation mode of Orai1 and Orai3 depending on the estrogenic status and/or the histological origin [21,22,24]. Orai3 contributes to the SOCE in luminal cells expressing estrogen receptors (ER^+^) such as MCF-7 and T47D cell lines, but not in the estrogen-negative (ER^−^) basal cells such as MDA-MB-231 [24], while Orai1 mediates SOCE in basal-like BC cell lines [22,24]. However, Orai1 still regulates SOCE in ER^+^ BC cell lines such as MCF-7 [21].

Orai1 is the most studied channel among the store-operated channels (SOC). Some studies have shown a modulation of the Ca^2+^ entry through Orai1, which does not depend on the Ca^2+^ store depletion. Indeed, in 2010, Feng et al. demonstrated another mode of activation of the Orai1 channels [10]. In luminal ER^+^ cells, Orai1, which is activated independently from STIM1, regulates basal Ca^2+^ entry and Ca^2+^ homeostasis. This mechanism involves the SPCA2 pumps initially located in the Golgi apparatus [10]. It has been demonstrated by co-immunoprecipitation and pull-down techniques that SPCA2 via its amino-terminus (N-ter) physically interacts with Orai1 at the level of the plasma membrane, which results in the activation of Orai1 by SPCA2 carboxyl-terminus (C-ter) and thus in an increase in the basal Ca^2+^ concentration [10]. Interestingly, the SCPA2/Orai1 coupling has also been shown in a cell model of lactation. Indeed, SPCA2 and Orai1 were found co-localized in mouse lactating glands and participate in a SICE to support lactation [25]. Therefore, it seems that BC cells redirect this SPCA2-dependent Orai1 activation to acquire cancer capacities. SICE induced by the Orai1/SPCA2 coupling has also been shown in the MCF-7 cell line, where it regulates cell proliferation [10]. Moreover, our team reported that SPCA2 also constitutes a complex with Kv10.1 potassium (K^+^) channels in ER^+^ cell lines and allows its trafficking from the Golgi to the plasma membrane [26]. Both SPCA2 N-ter and C-ter are involved in this trafficking [27]. Indeed, in MCF-7 cells, SPCA2 regulates the localization and the activity of both Kv10.1 and Orai1 channels, mediating a SICE able to sustain channel membrane localization and Erk1/2 phosphorylation, and to promote cell survival in a collagen environment [26,28].

As seen above, when a store-independent activation of Orai1 occurs, other proteins are involved to form a functional or physical complex with Orai1. It is the case in potassium channels. K^+^ channel activity permits K^+^ ion efflux that induces a membrane potential hyperpolarization, therefore, increasing the driving force for Ca^2+^ that favors Ca^2+^ entry in the cell [29]. Both the voltage-gated Kv10.1 and the small-conductance Ca^2+^-activated potassium channel (SK3) were found to work in partnership with Orai1. Indeed, these K^+^ channels, by regulating the membrane potential, regulate Ca^2+^ entry in basal-like BC cells.

For both of these channels, it has been found that Orai1 was the main actor in the constitutive Ca^2+^ entry in BC. In fact, both Kv10.1 and SK3 functionally regulate the Ca^2+^ entry through Orai1 leading the cell migration regulation. Indeed, Kv10.1 was observed expressed alongside Orai1 in invasive breast tumors and lymph node metastasis, and regulates cell migration through an Orai1-dependent constitutive Ca^2+^ entry [30]. On the other hand, it has been shown that SK3 knockdown inhibits BC bone metastasis [31]. This process is explained by the fact that, in the basal MDA-MB-435S cell line, Orai1 is recruited with SK3 to the lipid rafts, and following the SK3-dependent hyperpolarization, Orai1 is activated in a store-independent manner. Moreover, the same team showed an involvement of the SigmaR1 protein in the activity and localization of SK3 in lipid rafts [32]. The SICE through Orai1 activates the calpain leading to cell migration in the MDA-MB-435S cell line [31,32].

## 3. TRP Channels

The transient receptor potential (TRP) channels family is expressed and functional in most of the non-excitable cells in the human organism. They are known to be activated by various stimuli, such as growth factors, temperature, ligand, or mechanical stimuli, and are known to be abnormally expressed in cancers and especially BC. Many TRP channels have been identified as being up-regulated in BC and can be correlated with clinical parameters in BC. This is the case, for example, for TRP canonical (TRPC) channels (TRPC1, TRPC3, and TRPC6), TRP melastatin (TRPM) channels (TRPM6, TRPM7, and TRPM8), and TRP vanilloid (TRPV) channels (TRPV1, TRPV2, TRPV3, TRPV4, TRPV5, and TRPV6) [33,34,35,36,37,38]. In addition, it has been shown that TRPC6 is upregulated in BC cell lines and patient tissue compared to normal breast cell lines and healthy tissue, respectively [39]. However, TRPC6 expression is not correlated with tumor grades, estrogen receptor expression, or lymph node-positive tumors. Furthermore, the overexpression of TRPC1 has been discovered to be correlated with small tumors low proliferating (Grade 1), whereas TRPV4 is correlated with tumor grade, tumor size, and patient overall survival [40]. Moreover, given the estrogen-dependent trait observed in some BC, the TRPM8 channel has been shown to have its expression regulated by estrogen. Indeed, the application of estrogen on the MCF-7 cell line increases the expression of TRPM8 [33]. Both the role and expression of TRPM7 have been shown to depend on estrogen and/or aggressiveness status. Indeed, in ER^−^ tumors (not expressing the estrogen receptor and known to be the most aggressive), TRPM7 appears to be overexpressed in the most invasive areas [41], correlated to cancer metastasis and invasive BC [42], and regulates migration [41]. While in ER^+^ tumors, TRPM7 is overexpressed in the non-invasive areas, but at a lower level than in the invasive areas, and regulates cell proliferation [37].

In addition, it has also been shown that the TRPC1 channel expression is regulated via activation of the calcium-sensing receptor (CaR) [43]. Indeed, the activation of CaR by extracellular Ca^2+^ (up to 10 mM) increases TRPC1 expression, via the phospholipase C (PLC) and Erk1/2 pathway in MCF-7 cells [43,44,45]. Furthermore, TRPC1 is required for Erk1/2 phosphorylation and Ca^2+^ entry, and also for the proliferative effect induced by the activation of CaR. Moreover, the involvement of TRPC1 in the CaR-induced proliferation has been suggested [43].

Interestingly, TRPC1 expression is upregulated in hypoxia compared to normoxia conditions through HIF1α expression due to the presence of HIF1α and HIF1β binding domains in the TRPC1 gene promoter [46]. Silencing of HIF1α, but not HIF1β nor HIF2α, reduced TRPC1 expression. In basal BC cells under hypoxia, TRPC1 contributes to a basal Ca^2+^ entry and an increase of intracellular Ca^2+^ concentration in a constitutive manner. Indeed, TRPC1 silencing reduces the constitutive Ca^2+^ entry in cells grown under a hypoxic environment [46]. Moreover, the same study showed that TRPC1 promotes the epithelial-to-mesenchymal transition (EMT) in hypoxic conditions with an upregulation of the Snail1 mesenchymal marker via HIF1α signaling and downregulation of the epithelial maker Claudin-4 [46]. In addition, TRPC1 positively regulates the expression of the autophagy maker LC3BII through activation of the epidermal growth factor receptor (EGFR) as well as signal transducers and activators of transcription 3 (STAT3) phosphorylation during hypoxia [46].

In BC, Lee et al. have shown that TRPV4 calcium activity is required in cell migration via activation of the Akt signaling pathway as well as a downregulation of E-cadherin protein expression. In this study, they showed that activation of the upregulated TRPV4 by 4-α-phorbol-12,13-didecanoate (4α-PDD) triggers a Ca^2+^ influx responsible for the Akt signaling pathway and a sustainable phosphorylation of FAK which could be via the Akt signaling pathway. Moreover, Akt activation is responsible for a downregulation of β-catenin and E-cadherin [40]. Moreover, the activation of TRPV4 induces an EMT in MDA-MB-468 cells by increasing the expression of EMT markers, such as Vimentin, AXL, Serpin1, Twist, Snail, and CD44/CD24 ratio. In contrast, TRPV4-silenced cells presented a reduced single-cell motility but no change in the EMT markers’ expression [47].

The melastatin family of TRP channels is also a well-known regulator of carcinogenesis processes. For example, our team showed that TRPM7 is a key regulator in BC progression. It participates in cell proliferation as well as cell migration and invasion [37,41,42]. First, it has been found that TRPM7 silencing decreases the constitutive Ca^2+^ entry and hence the cell viability [37]. Guilbert et al. established that TRPM7 basal activity regulates ER^+^ BC cell line progression. In addition, they demonstrated that TRPM7 silencing decreased both Ca^2+^ entry and MCF-7 cell line proliferation [37]. However, it has been shown that TRPM7 regulates MDA-MB-231 cell line migration via its catalytic kinase domain, and not through its channel activity, by regulating the myosin II-based cytoskeletal tension and thereby SRY-Box transcription factor 4 (SOX4) [41,48,49]. Furthermore, research work on the MDA-MB-435S cell line showed that TRPM7 knockdown decreases both cell migration and invasion following a decrease in the MAPK protein phosphorylation [42]. However, this study does not show a direct channel activation of TRPM7, particularly when TRPM7 presents a kinase-type catalytic domain [42].

Another TRPM family member, the Ca^2+^-permeable TRPM8 channel, was found as a regulator of BC processes. This channel was shown to be activated in BC cells and associated with an elevation of cytosolic Ca^2+^ concentration following the application of icilin (TRPM8 agonist) [33]. However, the estrogen status does not seem to be involved in the TRPM8 activation state since 17β-estradiol increased TRPM8 mRNA expression but failed to affect the Ca^2+^ entry [33]. Moreover, it has been shown that TRPM8, following menthol or icilin activation, regulates BC cell proliferation and migration via activation of AMP-activated protein kinase–Unc-51 like autophagy activating the kinase 1 (AMPK-ULK1) signaling pathway, suggesting that TRPM8, by regulating the autophagy, leads the proliferative and migratory processes [50].

## 4. Voltage-Gated Calcium Channels

A number of studies have focused on the role of VGCC, which could be activated under normal cell culture conditions. Indeed, the resting membrane potential, measured by whole-cell patch-clamp technique, varies from −40 to −20 mV in BC cell lines [51,52,53]. The opening of VGCC at rest allows, therefore, a basal Ca^2+^ entry. Some VGCC see their expression and activity being altered in BC. This is the case in T-type Ca^2+^ channels, such as Cav3.1, Cav3.2, which are overexpressed in BC tissue [54,55]. Indeed, through an experimental and informatic study using microarray analysis, it has been found that certain L-type channels, such as Cav1.2 and Cav1.3, seem to be overexpressed in different types of cancer, including BC, and participate in inward Ca^2+^ entry following melatonin and 5α-dihydrotestosterone perfusion [56,57,58].

L-type VGCC were found to be active at a basal level and regulated by the L-type voltage-gated calcium channel γ 4 subunit (CACNG4) [59]. CACNG4 modulates L-type VGCC basal activation, and thereby the downstream processes. This subunit has been found to be involved in BC cell proliferation, motility, and adhesion. Its silencing reduced these cellular processes and its overexpression increased the metastasis to the lungs in vivo. Treatment with L-type channels antagonists Verapamil and Amlodipine decreased the MCF-7 and MDA-MB-231 cell proliferation. It has also been shown that CACNG4 silencing led to an increase in Ca^2+^ entry. However, the application of L-type channel antagonists decreased Ca^2+^ entry. These results suggested that CACNG4 subunit regulates the channel in an active state resulting in the higher intracellular Ca^2+^ concentration leading in fine to the inhibition of processes such as cell proliferation, motility, and adhesion [59]. Moreover, L-type VGCC are involved in BC cell invasion [60]. The activation of the L-type Ca^2+^ channel with a specific agonist BAY K8644 leads to an increase in the intracellular Ca^2+^ concentration responsible for filopodia stability. Indeed, treated cells with L-type channels pharmacological blockers, such as amlodipine besylate, felopidine, manidipine dichloride, and cilnipidine, lose their stable filopodia. Furthermore, it has been shown in the same study that integrin activation promotes filopodia formation through the proto-oncogene tyrosine-protein kinase Src signaling pathway, calpain activity, as well as a Ca^2+^ entry at the filopodia level. In addition, the L-type Ca^2+^ channel seems to be colocalized with myosin X (MYO10) within filopodia [60].

T-type Ca^2+^ channels, active at membrane potential from −50 mV and above [61], have been reported to regulate BC cell proliferation. Selective knockdown or pharmacological targeting to inhibit T-type Ca^2+^ channels reduced MCF-7 cell proliferation without altering cell viability [62]. A similar study was conducted and showed that treatment with mibefradil and pimozid (Ca^2+^ channel antagonists) inhibited T-type Ca^2+^ currents and reduced cell proliferation. However, due to the poor selectivity of the pharmacological blockers used, it has been concluded that T-type Ca^2+^ channels are involved in the cell proliferation alongside other VGCC, such as L-type Ca^2+^ channels [61].

## 5. Conclusions

**Figure 1:**
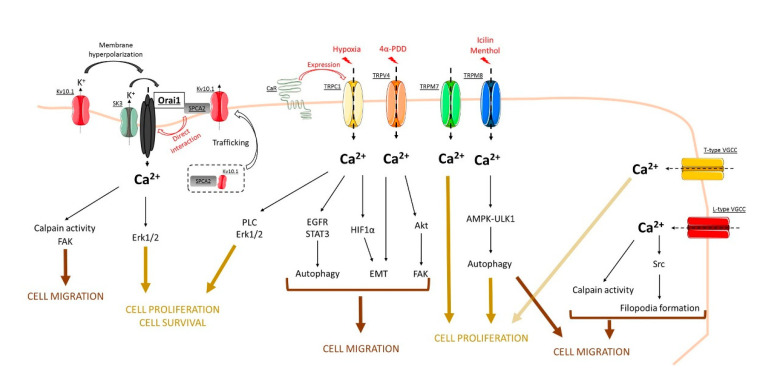
Concluding model that summarizes the role of store-independent calcium entry (SICE) through Orai, TRP, and voltage-gated calcium channels in the regulation of breast cancer processes. See, in the text, the role of each channel in cell processes such as cell proliferation, cell survival, as well as cell migration.

To summarize, Ca^2+^ channels are a protein family activated by a plethora of stimuli from store-dependent activation to the basal-activated state in BC. Based on studies demonstrated in this review, it seems that Ca^2+^ signaling is far from being fully understood in BC. As we schematized, the SICE constitutes a key effector in a multitude of BC cell processes, even though it is not the major actor in the Ca^2+^ flow as SOCE (see Figure 1). Nevertheless, further studies on Ca^2+^-regulated cell processes in BC should bring answers regarding the relationship and/or the complementarity between the role of SOCE and SICE in the regulation of BC progression via Ca^2+^ signaling.

## Abbreviations

AA	Arachidonic acid;
AMPK-ULK1	AMP-activated protein kinase–Unc-51 like autophagy activating kinase 1;
BC	Breast cancer;
Ca^2+^	Calcium;
CaR	Calcium-sensing receptor;
C-ter	carboxyl terminus;
EGFR	Epidermal growth factor;
EMT	Epithelial-mesenchymal transition;
ER^+^	Estrogen receptor positive;
ER^−^	Estrogen receptor negative;
Erk1/2	Extracellular signal-regulated kinases 1 and 2;
FAK	Focal adhesion kinase;
HIF1α	Hypoxia-inducible factor 1 α;
Kv10.1	Voltage-gated potassium channel;
LTC4	AA metabolite leukotriene C4 (LTC4);
miRNA	Micro-ribonucleic acid;
N-ter	Amino terminus;
PLC	Phospholipase C;
SICE	Store-independent calcium entry;
SK3	Small conductance calcium-activated potassium channel;
SOC	Store-operated channels;
SOCE	Store-operated calcium entry;
SOX4	SRY-Box transcription factor 4;
SPCA2	Secretory pathway calcium-ATPase;
Src	Proto-oncogene tyrosine kinase Src;
STAT3	Signal transducers and activators of transcription 3;
STIM	Stromal interaction molecule;
TRP	Transient receptor potential;
TRPC	Transient receptor potential canonical;
TRPM	Transient receptor potential melastatin;
TRPV	Transient receptor potential vanilloid;
VGCC	Voltage-gated calcium channel;
4α-PDD	4-α-phorbol-12,13-didecanoate.
